# Is depression a global brain disorder with topographic dynamic reorganization?

**DOI:** 10.1038/s41398-024-02995-9

**Published:** 2024-07-05

**Authors:** Georg Northoff, Dusan Hirjak

**Affiliations:** 1 Mind, Brain Imaging and Neuroethics Research Unit, The Royal’s Institute of Mental Health Research, University of Ottawa, Ottawa, ON Canada; 2 Department of Psychiatry and Psychotherapy, Central Institute of Mental Health, Medical Faculty Mannheim, University of Heidelberg, Mannheim, Germany; 3 German Centre for Mental Health (DZPG), Partner Site Mannheim, Mannheim, Germany

**Keywords:** Molecular neuroscience, Pathogenesis

## Abstract

Major depressive disorder (MDD) is characterized by a multitude of psychopathological symptoms including affective, cognitive, perceptual, sensorimotor, and social. The neuronal mechanisms underlying such co-occurrence of psychopathological symptoms remain yet unclear. Rather than linking and localizing single psychopathological symptoms to specific regions or networks, this perspective proposes a more global and dynamic topographic approach. We first review recent findings on global brain activity changes during both rest and task states in MDD showing topographic reorganization with a shift from unimodal to transmodal regions. Next, we single out two candidate mechanisms that may underlie and mediate such abnormal uni-/transmodal topography, namely dynamic shifts from shorter to longer timescales and abnormalities in the excitation-inhibition balance. Finally, we show how such topographic shift from unimodal to transmodal regions relates to the various psychopathological symptoms in MDD including their co-occurrence. This amounts to what we describe as ‘Topographic dynamic reorganization’ which extends our earlier ‘Resting state hypothesis of depression’ and complements other models of MDD.

## Introduction

### Co-occurrence of symptoms and brain-symptom relationship

**Fig. 1:**
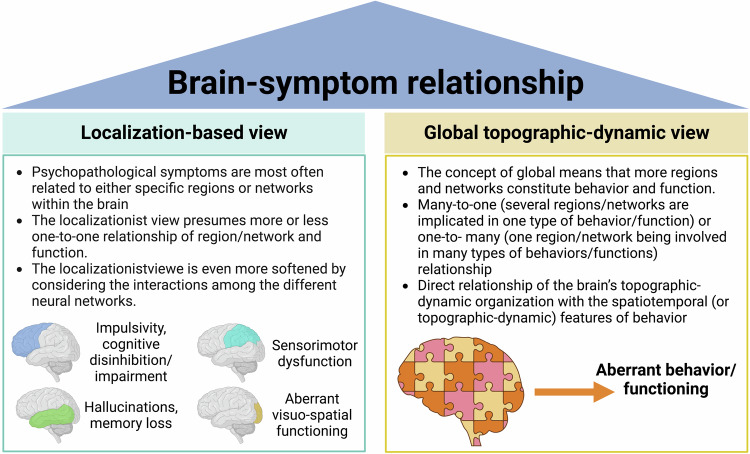
Major aspects of the localization- and global-based views on the brain-symptom relationship.

Major depressive disorder (MDD) is considered a mood disorder with symptoms like low mood, continuous sadness, lack of drive, and loss of interest or pleasure [1]. However, the range of symptoms extends far beyond as these patients also exhibit co-occurring cognitive changes (e.g., rumination [2] and impaired working memory [3]), somatic neurovegetative alterations (chest pain, constipation, irritable bowel or other [4]), interpersonal (e.g., social anxiety and withdrawal symptoms [5]), sensory-perceptual deficits (aberrant visual perception [6]), and motor or psychomotor retardation or agitation [7]) symptoms. Where and how do such a multitude of symptoms and thus their co-occurrence come from? Addressing this question is the main goal of our paper. Before we describe our hypothesis and approach, we would like to take a brief look at two ways of connecting the brain and symptoms in MDD, e.g., localizationist and globalist view (Fig. 1).

According to the so- called localizationist view of the brain, psychopathological symptoms are related to either specific regions or networks within the brain in more or less a one-to-one way. For instance, abnormal sadness has been related to increased activity in the subgenual anterior cingulate cortex [8, 9] and the amygdala [10–12]. Rumination [13, 14] and especially increased self-focus [15–19] are associated with increased activity in the cortical midline structures of the default-mode network (DMN). Somatic neurovegetative symptoms are related to the insula [4] and psychomotor retardation is often associated with decreased activity in the motor cortex [7, 20]. Finally, working memory deficits are related to hypofunction of the lateral prefrontal cortex [3]. An extension of such a local region or network-based approach can be found in the recent mapping of an MDD circuit which supposedly underlies all MDD symptoms [21, 22].

Although the localizationist approach to the brain function relationship has contributed a great deal of knowledge about mechanisms of mental disorders, the current methodological developments show that the brain function relationship may operate in a more hierarchical and holistic and thus global way. We shall note that we do not see our more global approach in contradiction to the more local views that focus more on regions, networks, and specific frequencies. Rather, we assume that activations in specific regions, networks, or frequencies may need to be conceived in a broader context, that is, they may emerge and differentiate from each other from their shared underlying global topographic dynamic basis [21, 22]. Conceived in this way, our global topographic dynamic approach may provide the broader neural background layer for the specific regions, networks, and frequencies including their respective function operating as foreground layers [21, 22].

Recent evidence showed that a much more global view of the brain-behavior relationship comes more and more to the forefront. For instance, the brain seems to exhibit an intrinsic organization, e.g., topography along the axis of unimodal and transmodal regions. Such unimodal transmodal gradient is reflected in that higher-order associative transmodal regions exhibit much stronger functional connectivity patterns among themselves than with the lower-order sensory unimodal regions [23, 24]. This goes along with corresponding dynamic differences as the transmodal regions show longer timescales than the unimodal regions [25–29].

Such a global topographic view of the brain makes it possible that one and the same function can be associated with several regions or networks, e.g., many to one relation, or, alternatively that one region or network can be involved in different functions, e.g., one to many relations [30]. Correspondingly, recent animal studies showed that behavior like motor action is indeed mediated not only by the motor cortex itself and related regions but basically by the whole brain featured by its various regions and different timescales [31–33]. On the human side global brain activity is often measured in fMRI by the global signal (GS) and its representation in specific regions or networks, e.g., global signal topography (see ref. [34] for a recent review).

In particular, recent evidence indicated that the brain exhibits ‘global’ activity, similar to economic and climate systems, modulating non-uniformly across local regions and networks [23, 24]. This modulation involves subcortical nuclei like the raphe nucleus, nucleus basalis meynert, and substantia nigra affecting cortical activity [35, 36]. Animal studies suggested multiple brain regions contribute to specific behaviors, supporting the role of global brain activity in behavior. In human fMRI, global activity is measured by the GS, defined as the average of whole-brain voxels or gray matter voxels [37].

It should be mentioned that GS is often considered in a primarily methodological way given that its inclusion or exclusion significantly impacts the relationship between task-positive and task-negative networks, potentially introducing anti-correlation [38]. Moreover, GS has been linked to extra-neuronal sources such as respiration [39] and heartbeat [40] which, though, may modulate the brain’s global activity in a physiological way [39]. This is further supported by combined ECoG/electrophysiology and fMRI studies that revealed a direct relationship between GS and neuronal activity, suggesting GS is not merely noise [41, 42]. Taken in such a physiological way, GS shows a particular dynamic topography, with variations in different brain regions, and alterations in various neurological and mental disorders [41, 42], indicating its potential physiological role in the brain and behavior.

### Goal and specific aims

We here apply such a more global topographic approach to MDD showing how it can very well account for the observed topographic changes and the co-occurrence of symptoms. We will do this in three stages: First, we will present a narrative review of global topographic changes in MDD with a specific focus on the changes in the unimodal transmodal topography. Second, we will focus on two candidate mechanisms, namely changes in dynamics and excitation-inhibition balance, which may underlie and mediate the abnormal topographic organization in MDD. Finally, we seek to connect such topographic dynamic reorganization to the various symptoms of MDD including their co-occurrence.

Before going ahead, we shall briefly determine two of our key concepts, namely global and topography. Rather than in an absolute sense (with all single regions recruited during specific functions), the concept of *global* is here meant in a relative and more quantitative sense, that is, more or less regions and/or networks are involved in constituting behavior and function [34]. The brain-behavior/function (or brain-symptom) connection can then be modeled in terms of many-to-one (several regions/networks are implicated in one type of behavior or function) or one-to-many (with one region/network being involved in many types of behaviors) relationships [30, 43]. Neural activity changes in a specific region/network may thus at best be necessary but not sufficient by themselves for the symptoms. Instead, the more global topographic relationships among the different regions or networks are then sufficient for behavior, function, and symptoms.

The concept of *topography* comes originally from geography where it is concerned with the organization and structure of natural features like mountains, lakes, forests, etc. including especially how they relate to each other—this is, for instance, visualized on maps. Topography in this sense needs to be distinguished from the concept of topology which refers to a branch of mathematics that focuses on shapes and their distortions [44]. We here focus on the brain’s topography rather than its topology as we target the organization and structure of the various regions and networks among each other, that is, how the balances and interdependencies among especially unimodal and transmodal regions are shifted in an abnormal way in their neural activities in MDD. Together, this amounts to what we describe as ‘Topographic reorganization’ which extends our earlier ‘Resting state hypothesis of depression’ [45] and complements other models of MDD (see Box 1).

BOX 1 Relationship to other models of MDD There are various models of MDD (see Marx et al. [109]). We early formulated the resting state hypothesis of MDD (Northoff et al. [45] which, in a nutshell, associates the resting state’s activity and its networks with the different symptoms. Given that the topographic dynamic reorganization is a key feature of the brain’s spontaneous activity, e.g., its resting state, the here suggested model can be seen as a specification and extension of our earlier resting state model. Moreover, the resting state’s topographic subcortical cortical reorganization naturally converges with the monoaminergic models and especially the serotoninergic model [109]. The topographic model would conceive circuit models [22], that pursue an RDoC-like approach [110, 111] with a focus on selected connected subcortical and cortical regions, as secondary manifestations of underlying more primary global topographic changes that encompass both subcortical and cortical levels. Models involving both brain and body like neuroendocrine, neuroinflammatory, stress with the hypothalamic adrenal axis, and gut microbiome [109], may all converge on the brain’s spontaneous activity and thus its resting state (Northoff et al. [45] where these more physiological changes may elicit the above described topographic changes. Hence, future studies may want to link the here suggested subcortical cortical topographic dynamic reorganization with the endocrine, inflammatory, and other measures postulated in these models.

One influential computational hypothesis is the predictive coding model of mood and MDD where mood states are seen as higher-order priors that reduce uncertainty about lower-order interoceptive and exteroceptive states [112–114]. Converging the predictive model with the topographic account, we suppose that the abnormally high synchronization of default-mode network regions with themselves and other regions of the brain exerts an abnormally strong top-down prediction on lower-order sensory and motor regions [112–114].

Moreover, given that the same regions exhibit very long timescales, it may explain the long-lasting effect of these predictions and their overriding or enslavement of the much shorter and faster lower-order motor action and sensory prediction. Merging both predictive and topographic accounts, one may want to speak of an abnormally strong synchronization of higher predictions with lower-order prediction errors. The letter “loose” its partial independence from the former as they are no longer properly modulated by external inputs themselves due to the abnormally strong synchronization of sensorimotor regions by the transmodal regions’ predictions.

Converging the predictive approach with interoception, Barrett et al. [115] highlighted MDD as a disorder of allostasis reflecting the brain’s abnormal control and prediction of the body’s metabolic and energetic regulation. This, according to Barrett et al. [115], results in the sensory input regions being basically shut off resulting in a somewhat “locked-in brain” where the external inputs as mediated by the sensory regions are no longer properly processed. Needless to say, that converges well with the decreased and abnormally slow activity of the sensory and motor regions described above. Interestingly, as shown in the cited article, the regions modulating allostatic interceptive prediction involve more or less the same subcortical and cortical limbic regions that are also mostly affected by the topographic dynamic reorganization. This suggests convergence of the allostatic energetic model with the here suggested topographic dynamic reorganization something which is further supported by a recent article linking timescales and allostasis [112–114].

Finally, yet another approach is the phenomenological model of MDD which focuses on the subjective experience highlighting the abnormal slowness and the absence of changes due to the stalling of time in the past [92, 93, 116]. This is well compatible with especially the dynamic features of the topographic reorganization that shows decreased speed with slowing down on both neural and affective cognitive levels as described above. The reduced dynamics may then be considered a basic disturbance that underlies the topographic changes and ultimately the various depressive symptoms which are then conceived primarily spatiotemporal alterations as suggested in Spatiotemporal psychopathology [95, 98–100].

## Part I: Global topographic changes in MDD

### Abnormal global signal topography in both default-mode network and sensory regions

There are various lines of evidence for global changes in the brain’s neural activity in MDD (see Box 2 for relevant measures of brain's global activity and its dynamics). One way to investigate global changes is to measure the global signal and its distribution or representation within specific regions, e.g., global signal topography (see [34] for review). The global signal is investigated by the brain’s global functional connectivity which, physiologically, is based on neural synchronization among the different regions [34, 46, 47]. Changes in global functional connectivity thus indicate changes in neural synchronization. Han et al. [48] showed decreased static global functional connectivity in parahippocampus and hippocampus in acute MDD subjects which negatively correlated with the degree of psychomotor retardation, e.g., less global functional connectivity in these regions, the more psychomotor retardation. Moreover, MDD subjects exhibited increased dynamics, e.g., variability in the medial prefrontal cortex. Niu et al. [49] investigated 71 acute MDD subjects and 71 healthy controls (HC) with fMRI analyzing their whole-brain static and dynamic functional connectivity. Using machine learning as the classifier, they showed that dynamic FC both with and without global signal allows for 1oo% classification of MDD vs HC. DMN, cerebellum, and subcortical regions were among those to best differentiate MDD and HC. In contrast to dynamic FC, static FC allowed for high discrimination only when no GSR was conducted (96% while the inclusion of GSR decreased the rate to 38%.

Together these findings clearly highlight the key role of global brain activity and its dynamics for predicting neural differentiation of MDD vs. HC. Further, Liu et al. [50] investigated graph theoretic global network properties in unmedicated MDD (*n* = 34) and anxiety disorder (AD) (*n* = 31). They showed that both groups showed major changes in global network properties and also local more nodal features. While the classification accuracy of MDD and AD reached 71%, there was a high prediction of symptom severity by the global dynamic subcortical cortical FC measures in both groups. Thus, the combination of both global and local changes suggests a multilayered topographic pattern in MDD as the authors highlighted in the title of their paper.

Furthermore, Scalabrini et al. [51] demonstrated increased representation of GS specifically the DMN in MDD compared to HC. Moreover, the increased GS in DMN was abnormally correlated with GS in all other non-DMN regions in MDD whereas their relationship remained independent, e.g., non-correlated in HC [51]—the DMN thus seems to “enslave” the non-DMN networks in MDD. Finally, although the subject number was quite low with about 4o MDD, the degree of DMN-non-DMN FC, i.e., the abnormal GS topographic representation, could predict clinical symptoms to a high degree, i.e., 90%, as revealed in vector machine learning. This underscores the potential diagnostic relevance of global brain activity. These findings are further supported by observations of increased global resting state functional connectivity indexing higher degrees of neural synchronization between sensory and DMN regions in MDD [29].

**Fig. 2:**
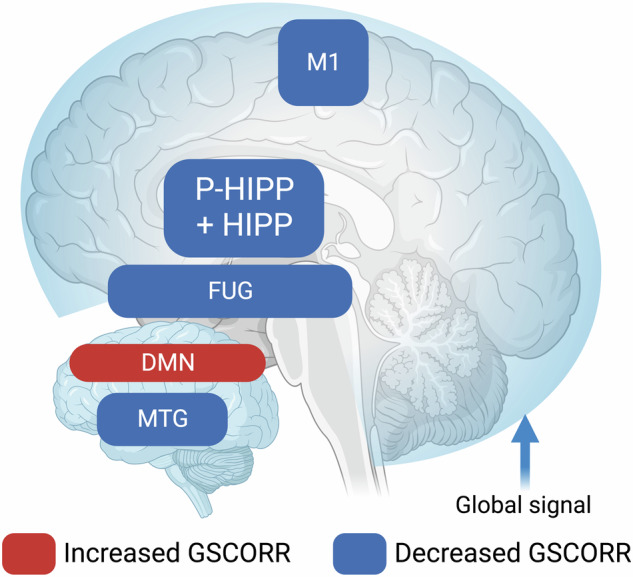
Altered global signal topography in MDD. The transparent bonnet represents a global signal being differently distributed across different regions/networks. GSCORR task-related global signal correlation, P-HIPP parahippocampus, HIPP hippocampus, DMN default-mode network, MTG middle temporal gyrus, FUG fusiform gyrus, M1 primary motor cortex.

The study by Lu et al. [52] extended the investigation of global signal topography (GS topography) from the resting state to task-related conditions, focusing on global signal correlation (GSCORR) during specific tasks. Their findings revealed that unmedicated patients with MDD exhibited decreased task-related GSCORR in the DMN and premotor cortical regions when exposed to negative and fast-presented visual stimuli, compared to slow and neutral stimuli [52]. Additionally, the study observed a significant correlation between the changes in task-related GSCORR in both anterior and posterior DMN regions, as well as the premotor cortex, and the severity of psychomotor retardation in MDD patients [52]. Specifically, lower GSCORR in these areas was associated with higher degrees of psychomotor retardation [52]. Taken together, the dysfunction in transitioning from unimodal to transmodal regions, coupled with the altered global signal dynamics, underpins many of the cognitive and emotional disturbances observed in MDD. These neural characteristics provide a more comprehensive picture of the disorder’s pathophysiology, emphasizing the importance of considering both local and global brain dynamics in understanding and treating MDD rather than BD or SZ (Fig. 2). This holds the promise of the future differential-diagnostic use of these uni-transmodal gradients in clinical settings for which larger scale studies including all three disorders are warranted.

BOX 2 Relevant measures of the brain’s global activity and its dynamics **GS Correlation (GSCORR):** GSCORR, the most accepted measure for GS topography, is calculated using Pearson’s correlation between the global signal (GS) and each voxel’s time series. This method reveals typical GS topography in healthy controls and altered topography in psychiatric disorders and loss of consciousness. Temporal dynamics of GSCORR, such as time lags between GS and local activity, have been studied in acute stroke.

**Global Functional Connectivity/Global Brain Connectivity (GFC/GBC):** GFC/GBC measures GS topography by averaging Pearson’s correlation between each voxel and every other voxel. Despite different calculations, GFC/GBC and GSCORR yield nearly identical spatial patterns when time series are normalized. GFC is useful in cognitive and clinical studies, though it is computationally intensive and lacks temporal information. GS regression can be performed with GFC to treat GS as noise, but this is not feasible with GSCORR.

**GS Regression (GSR):** GSR evaluates how GS regression impacts functional connectivity results. Recent studies analyze beta weights from GS regression, which mimic patterns from GSCORR and GFC, suggesting robust GS topography. However, direct comparisons between GS regression weights and other measures are lacking.

**Systemic Low-Frequency Fluctuations:** The GS may originate from systemic circulatory oxygenation fluctuations, indicating differential blood transit times in the cerebral neuronal vasculature. These low-frequency oscillations may be linked to the neuronal vascular connection through vasomotion, arterial CO2 fluctuations, or Mayer waves, while they are and are associated with arousal fluctuations.

**Autocorrelation Window (ACW):** This refers to the measurement of the correlation of the signal with itself over time. It is usually calculated by measuring the correlation of one and the same signal with itself across its different time points across different lags. The more the signal correlates with itself across more extended time lags, the longer the correlation with itself lasts and the longer the autocorrelation window. Hence, the autocorrelation window indexes the duration over which neural activity remains more or less the same over time.

### Global signal topography—monitoring therapeutic effects

One of the first fMRI studies on the neuronal effects of antidepressants was conducted by Van de Ven et al. [53] in 10 healthy volunteers. This study found that escitalopram does not modify the global functional structure of the DMN. Nevertheless, this study also showed a reduction in regional pairwise connectivity within the DMN, specifically between the anterior and posterior cingulate cortex, hippocampal complex, and lateral parietal regions due to escitalopram treatment. Later on, Eshel et al. [54] investigated 36 acute MDD subjects receiving either active or sham transcranial magnetic stimulation (TMS) of the dorsolateral prefrontal cortex (DLPFC). They showed that active but not sham TMS caused an increase in the global functional connectivity of the lDLPFC which, at baseline before TMS treatment, predicted treatment response. Moreover, functional connectivity from lDLPFC to the amygdala also changed during treatment. Together, these results suggested that global functional connectivity with regard to the dorsolateral prefrontal cortex allows predicting TMS treatment outcome in acute MDD.

Other fMRI studies investigated the impact of ketamine treatment on global functional connectivity in acute unmedicated MDD. Abdallah et al. [55] showed decreased global functional connectivity in the lateral prefrontal cortex at baseline, e.g., before treatment, which increased and “normalized” after a single infusion of ketamine treatment. The reverse pattern was observed in posterior cortical midline structures which showed increased global FC before ketamine and decreased global FC after treatment (see also [56]), though. Long et al. [57] observed increased whole-brain-rich club functional connectivity in MDD subjects undergoing cognitive behavioral therapy or fMRI feedback when compared to an MDD group without treatment. This is in line with Zhu et al. [58] who show that higher variability of GS in DMN regions leads to better treatment response to antidepressants in MDD.

Last but not least, the study by Zhang et al. [59] evaluated the effectiveness of combining ketamine with ECT in patients resistant to propofol-ECT for depression (ECT-RD). A total of 28 patients with ECT-RD received alternating treatments of ketamine and propofol-ECT over two weeks. The therapeutic impact was measured using the Hamilton Depression Scale, while brain function changes were assessed through global functional connectivity density (gFCD) and functional connectivity strength (FCS). Results indicated that adding ketamine improved treatment outcomes compared to propofol-ECT alone, particularly increasing gFCD in the left temporal and subgenual anterior cingulate cortex while decreasing FCS within the default-mode network. These functional connectivity changes lasted for 10 days whereas the clinical effects were sustained only for 7 days. These findings suggested that ketamine enhances the effects of propofol-ECT in ECT-RD patients, whose global neuronal and therapeutic effects seem to remain short-lived though. Taken all studies together, their results suggested the potential use of alterations in the brain’s global topography for therapeutic effects monitoring.

### Abnormal uni-/transmodal gradients in MDD

These findings highlighted an abnormal topographic shift of global brain activity from unimodal lower-order sensory and motor regions in their periphery to more transmodal higher-order associative regions in the core. This can be directly tested by testing and measuring the gradients between uni-/transmodal regions reflecting the core-periphery organization of the brain [23].

Recent studies show indeed abnormal uni-/transmodal gradients in MDD. Conducting a recent large-scale resting-state fMRI study by Xia et al. [20] showed that the uni-/transmodal gradient explains a lower variance of resting state signals of MDD than in healthy subjects. There was also less spatial variation as well as a shift of the uni-/transmodal gradient towards the DMN in MDD subjects, i.e., the gradient scores of the visual network and the SMN shifted towards the center (and thus away from the periphery towards the core). These findings are compatible with the observation that transmodal DMN activity is abnormally correlated with unimodal sensory activity [51].

Finally, using the Neurosynth platform for large-scale automated synthesis of fMRI data, Xia et al. [20] could show that the regions with altered gradients are related to those sensory-perceptual and cognitive functions that are changed in MDD [20]. Other studies confirm such abnormal unimodal transmodal topography. Pasquini et al. [60] observed lower degrees of dispersion with a reduced gradient of DMN, limbic and executive regions, and networks suggesting their increased neural synchronization. This was observed especially treatment treatment-resistant MDD patients and correlated with their baseline level of anxiety. Xiao et al. [61] also found similar changes in the uni-/transmodal gradient in MDD on the cortical level and even on the subcortical level (see below for details. Taken together all findings, these studies strongly suggest abnormal topographic shifts from unimodal sensory and motor regions to more transmodal regions like DMN and prefrontal cortex in MDD.

Finally, another study [62] suggested that these global topographic changes are related to changes in temporal features, e.g., dynamics. The rs-fMRI study by Javaheripour et al. [62] investigated the neural mechanisms underlying MDD by analyzing whole-brain dynamics using a hidden Markov model (HMM). This study involved a large sample of 314 patients with MDD and 498 HC. The HMM was employed to identify variations in functional connectivity and brain activity, defining six distinct states of neural dynamics. The findings reveal that compared to HC, individuals with MDD spend more time in, and show greater temporal stability within, a neural state characterized by reduced functional connectivity across all brain networks but increased activity in regions of the somatosensory motor, salience, and dorsal attention networks. This state’s prevalence and stability correlated significantly with the severity of depression symptoms. In contrast, HC predominantly exhibited a state with strong connectivity among regions but weaker overall global brain activity. These results highlight that disruptions in the temporal features of the brain’s global topography are linked to the persistence and severity of depressive symptoms in MDD.

## Part II: Neural mechanisms of the brain’s abnormal global topography

The above mentioned findings clearly indicated an alteration in the brain’s global activity in MDD with a shift from unimodal sensory regions to transmodal associate regions. We therefore ask where and how such a global topographic shift is coming from? We here identify two key candidate mechanisms, namely dynamic changes with abnormal slowing down (ABS) and abnormal excitation-inhibition balance (EIB).

## Mechanisms I: Slowing down with reduced dynamics in uni-/transmodal regions

The brain’s uni-/transmodal topography goes along with corresponding dynamic differences like in timescales. As measured by the intrinsic neural timescales (INT) through the autocorrelation window (ACW), studies in both fMRI [63, 64] and MEG [25, 26] show different lengths in the temporal windows of uni- and transmodal regions. Specifically, the unimodal regions exhibit shorter ACW while the transmodal regions exhibit longer ACW [25, 27, 28].

One would consequently expect that the topographic shifts from unimodal to transmodal regions in MDD go along with corresponding dynamic shifts from shorter to longer timescales. There is indeed initial evidence for that. Resting state activity is abnormally slow in the visual cortex and medial prefrontal cortex as well as in various other regions in MDD as evidenced by shifts in the power spectrum towards slower frequencies with decreased power in faster frequencies [65]. This is in line with other resting state findings of longer dwelling of dynamic functional connectivity with lower variability in DMN and frontoparietal regions thus indexing higher and longer temporal stability [29].

Together, these findings, albeit tentatively, suggest that the topographic shift from uni- to transmodal regions is accompanied by a corresponding dynamic shift from shorter to longer timescales in MDD. The origin and mechanism of such seemingly global slowing of cortical activity and its impact on the global topography remain yet unclear, though.

One possible hypothesis is that the abnormal slowing down in input regions like the visual cortex during the processing and encoding of fast input dynamics may reverberate throughout the whole brain and thereby shift its topography from uni- towards transmodal regions. There is indeed initial evidence for that. Slowing down of both resting and task state activity in the visual cortex goes along with increased functional connectivity, e.g., synchronization to DMN regions like the anterior cingulate cortex and the medial prefrontal cortex [12, 29, 52, 65] which, in turn, may slow down the latter. Specifically, the studies showed that dynamic changes, e.g., slowing down in the visual cortex resting state relate to its abnormal, e.g., increased functional connectivity with the regions in the anterior DMN [29]. Albeit tentatively, this suggests a close relationship between dynamic shifts with topographic changes in MDD. The dynamic shifts, e.g., slowing down in for instance visual cortex are further supported by the observation of decreased reactivity to especially fast visual stimuli in MDD subjects [52, 65]. We need to be careful, though as future studies are necessary to link the abnormal slow dynamics in visual cortex resting state dynamics to both unimodal transmodal topographic shifts and decreased reactivity to fast stimuli during states.

Yet another possible hypothesis is that subcortical dynamic changes in the raphe nucleus may reverberate to the cortex. There is indeed initial evidence that even subcortical regions like the serotoninergic raphe nucleus and the dopaminergic ventral tegmental area exhibit spectral slowing, e.g., shift of the power spectrum towards slower frequencies, with lower permutation entropy [66]. Given the known serotonergic modulation of especially the DMN [36], the raphe nucleus spectral slowing may also affect the DMN by rendering slower its neural activity. Such slowing of DMN neural activity may prolong its timescales and thereby increase both its internal synchronization and its synchronization with non-DMN regions in abnormally strong ways. That, in turn, may lead to the observed shift from unimodal to transmodal regions including their abnormally low neural response to especially fast stimuli.

## Mechanisms II: Changes in the excitation-inhibition balance (EIB) lead to changes in the unimodal transmodal topography

Findings in healthy subjects show that the unimodal transmodal topography is accompanied by corresponding gradients in the EIB [67–70]. Specifically, the level of excitation relative to inhibition increases from unimodal to transmodal regions. This carries major implications for MDD. Given the abnormal topographic shift from unimodal to transmodal regions in MDD, one would expect abnormally increases in excitation relative to inhibition especially the transmodal regions.

This is indeed in line with the observation of brain-wide changes in the GABAergic and glutamatergic levels of EIB in MDD. A recent review by Hu et al. [71] took a topographic perspective on postmortem studies. Inhibitory GABA-related genetic markers are decreased in DMN regions as well as in unimodal regions like the occipital cortex [6, 12] and motor cortex [71] which, in turn, shifts the EIB in these regions towards increased levels of excitation. Albeit tentatively, we therefore suppose that the topographic shift from unimodal to transmodal regions is related to corresponding shifts towards increased excitation in the EIB.

Decreased levels of inhibitory GABA-related markers can also be observed in subcortical limbic regions like the amygdala and others which, as on the cortical level, shifts the EIB towards increased excitation [71]. The increased subcortical excitation may then reverberate onto the cortical level where it may lead to analogous changes. That is also in line with the observation that topographic unimodal transmodal gradient changes on the cortical level co-occur with changes in subcortical limbic regions like the amygdala and others like the thalamus and caudate [61]. Albeit tentatively, this further supports the hypothesis that the abnormal subcortical cortical topography is related to corresponding changes in the subcortical cortical EIB with a shift towards excitation. This is, at best, a tentative and preliminary exploratory hypothesis as the detailed mechanisms of linking EIB, subcortical changes, and cortical topographic shifts are not known yet.

**Fig. 3:**
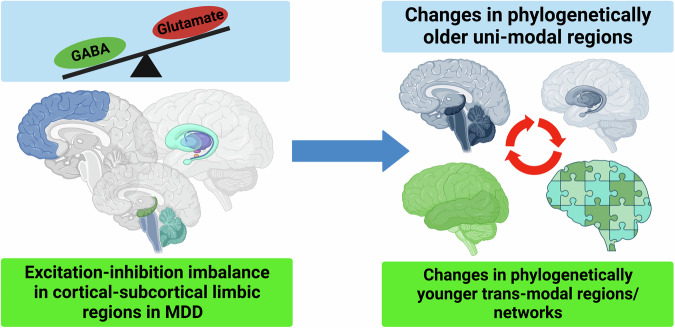
Changes in the excitation-inhibition balance (GABA vs. Glutamate) lead to changes in the unimodal and transmodal regions/networks.

Together, the findings suggest a global topographic shift in the EIB from unimodal to transmodal regions. Decreased GABAergic inhibition in sensory input regions and DMN regions seems to contrast with increased GABAergic inhibition in the lateral prefrontal cortex [71]. Moreover, the empirical data and computational modeling provided initial evidence that decreased GABAergic inhibition in sensory input regions like the visual cortex may be related to and, even stronger, drive the topographic shifts from unimodal to transmodal regions in MDD (Fig. 3). Applied to a clinical context, this means that therapeutic intervention with transcranial magnetic stimulation in visual cortex may change the abnormal topographic shift in MDD something which is indeed supported by two recent studies [72, 73].

Finally, albeit tentatively, reduced speed and altered EIB may be related to each other given the close relationship of increased levels of excitation in the EIB with longer temporal windows in the brain’s intrinsic neural timescales [67, 68]. When there is an imbalance, particularly an increase in excitation relative to inhibition, neural circuits may become hyperactive or overly synchronized, leading to slower overall dynamics. This is because excessive excitation without adequate inhibitory counterbalance can lead to prolonged neuronal firing and delayed signal termination, which manifests as slowed functional connectivity across regions and longer timescales within regions [71]. In particular, in MDD, the abnormal topographic shift and increased excitation in transmodal regions could abnormally prolong the latter’s timescales leading the abnormal slowness in both mood and cognitive processing including their abnormal stabilization and persistence. This remains speculative at this point, though.

## Part III: From brain to symptoms—topographic and dynamic reorganization connect neural and psychological levels

### Topographic reorganization—from ‘neural synchronization’ to ‘psychological synchronization’

The above mentioned findings clearly demonstrated topographic reorganization with a shift from unimodal to transmodal regions. The unimodal sensory regions are more strongly synchronized and thus connected with the transmodal regions of the DMN in the depressed than the non-depressed state [29, 51] with the former being “enslaved” by the latter [12, 29, 52]. We now suppose that such abnormally strong ‘neural synchronization’ of unimodal with transmodal regions is manifest in a correspondingly strong synchronization among the psychological functions mediated by unimodal and transmodal regions. Specifically, we assume that such abnormally strong ‘psychological synchronization’ concerns the relationships of 1. Perception and action with cognition and emotion, 2. Externally oriented cognition with internally oriented cognition, and 3. Non-self with self.

**Fig. 4:**
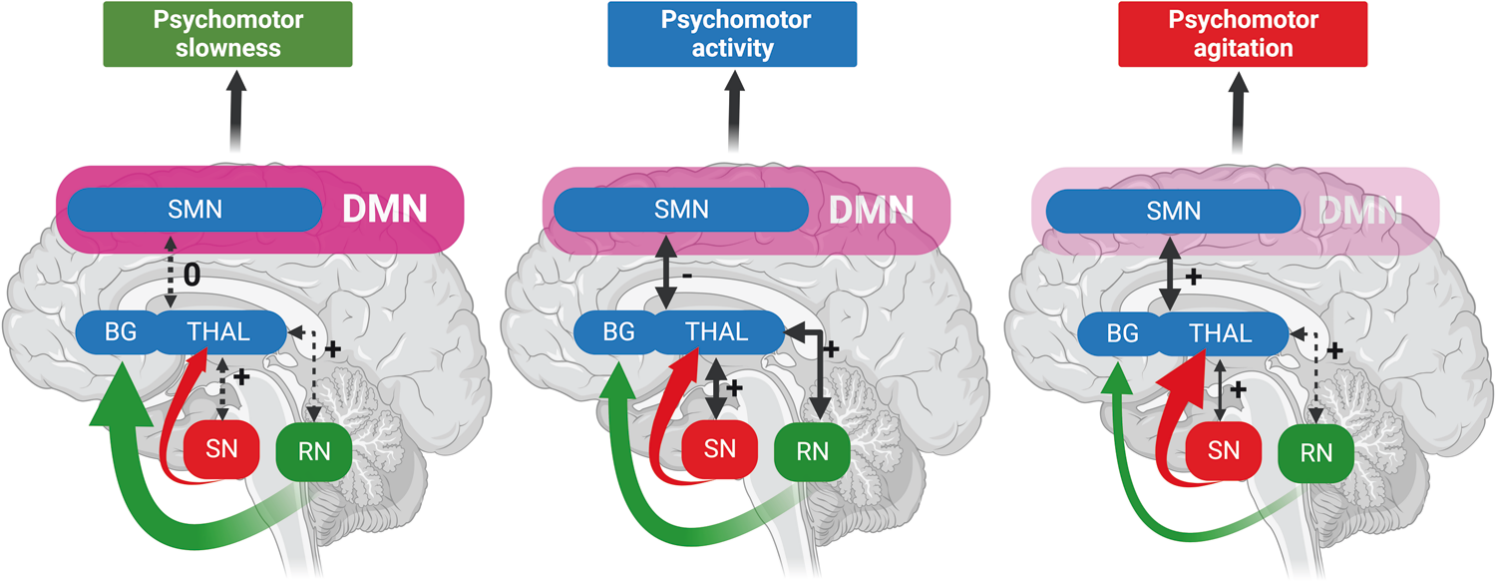
Subcortical-cortical layer and different psychomotor symptoms in MDD. Schematic representation of the relationship between functional connectivity (FC) between Thal-SMN, SN-BG/Thal, and RN-BG/Thal. Black arrows represent FC: thicker arrow represents high FC (independently from the sign), while dotted arrow low FC (toward zero values); “+” represents positive FC, “−” represents negative FC, “0” represents around zero FC. DMN in pink, transparency illustrates how strong the network is: weak in excitation and strong in inhibition. Green arrows illustrate the dopaminergic excitatory pathway from SN to THAL, the width of the arrow illustrates how strong the excitatory effect is. Red arrows illustrate the serotonergic inhibitory pathway from RN to BG (especially nucleus accumbens), and the width of the arrow illustrates how strong the excitatory effect is. DMN default-mode network, SMN sensorimotor network, BG basal ganglia, THAL thalamus, SN substantia nigra (dopamine), RN raphe nucleus (serotonin) (adapted according to ref. [107]).

There are various lines of evidence for a decrease in visual perception in MDD as being related to decreased visual cortex function [6, 52, 74–77]. Similar so with respect to motor function which is decreased, e.g., psychomotor retardation in MDD [20, 71, 78]. While psychomotor retardation goes along with decreased motor cortex activity [20, 78], the latter seems to stem from sources outside both the subcortical cortical motor regions like subcortical raphe nucleus [79] and cortical input regions like the visual cortex (Fig. 4) [12, 44, 52]. Correspondingly, these studies observe a correlation of psychomotor retardation with visual cortex function and its functional connectivity with the motor cortex rather than solely and exclusively with motor cortex activity itself (Fig. 4) [52, 71]. While perception and action are decreased in MDD, negative emotion, and cognition are abnormally increased. This is manifest in abnormal sadness and rumination which both have been associated with increased neural synchronization in DMN [10, 11, 13–15, 80].

**Fig. 5:**
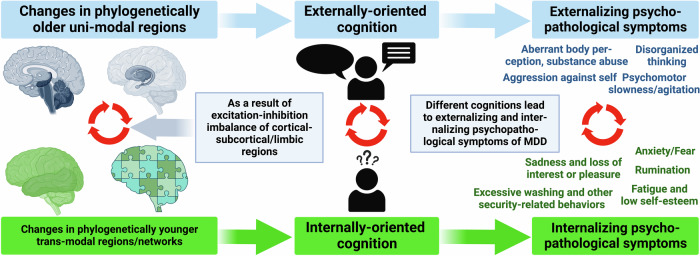
Uni- and transmodal brain regions and their alterations leading to externally- and internally oriented cognition and then different externalizing and internalizing psychopathological symptoms (HiTOP-based) and their co-occurrence (red arrow circle) in MDD.

We now assume that the degree of abnormally increased neural synchronization, e.g., functional connectivity, of the unimodal sensory and motor regions with the transmodal DMN regions [29, 51, 76] is directly related to the degree of increased ‘psychological synchronization’ of impaired visual perception and psychomotor retardation with abnormal increases in sadness and rumination. Analogously so for the balance of internally and externally oriented cognition as well as self and non-self. There are various lines of evidence that the balance of internally and externally oriented cognition is mediated by the topographic balance of unimodal and transmodal regions (Fig. 5) [81]. If the neural balance now shifts abnormally strongly towards the transmodal regions, there will be an analogous psychological shift towards internally oriented cognition. This is typically manifest in rumination [14], increased incidence of internally oriented thought contents [82], and increased self-focus [16–19]. Keskin et al. [19] could indeed show that abnormally increased global activity representation in the cortical midline regions of the DMN, as related to the self, results from increased synchronization of these regions with the rest of the brain including unimodal sensory and motor regions.

**Fig. 6:**
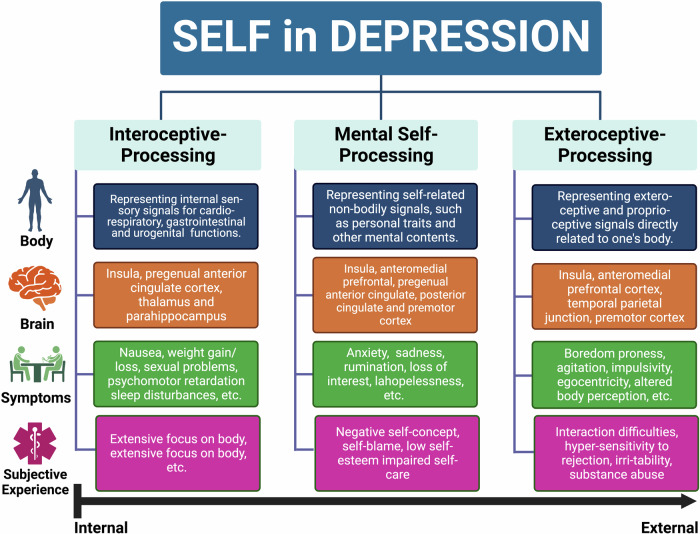
A schematic representation of the three-level-processing model of self in MDD (according to Qin et al. [108]) being associated with different brain regions and leading to different psychopathological symptoms (HiTOP-based) of MDD.

Together, these findings suggest that the brain’s topographic reorganization with increased ‘neural synchronization’ of unimodal with transmodal regions is manifest in and translates into corresponding ‘psychological synchronization’ of perception and action, externally oriented cognition, and non-self with emotion, internally oriented cognition, and self (Fig. 6). Accordingly, topographic reorganization with increased synchronization is manifest on both neural and psychological level in more or less analogous ways.

We need to be careful, though. The exact mechanisms of how neural synchronization translates into psychological synchronization remain yet unclear. One way to investigate that would be for instance to measure synchronization on the neural level in a dynamic way, that is, its fluctuations over time, and then to test how that relates to the synchronization among sensory, motor, affective, and cognitive symptoms. In addition to the time series on the neural connectivity among the different regions, obtained at multiple time points like over days and weeks, one would then want to measure the fluctuations in the correlations among the different symptoms at the same time points. Ideally, one would predict that the fluctuations in the neural synchronization among the relevant regions or networks involved in movement, mood, and cognition relate to the fluctuations in the relationship among the respective symptoms.

### Dynamic reorganization—from ‘neural slowness’ to ‘psychological slowness’

We saw that the topographic shift from unimodal to transmodal regions is accompanied by more or less corresponding dynamic shifts from shorter and faster to longer and slower timescales in the brain’s neural activity. We now suppose that corresponding temporal shifts are also manifest on the psychological level with for instance the manifestation of abnormal slowness in various psychological functions. This is indeed the case.

MDD is characterized by abnormal slowness with reduced speed on psychological, behavioral, and phenomenological levels. MDD subjects show reduced speed in their information processing, they are too slow which impairs their cognitive performance in tasks with time limits while they are normal in cognitive tasks without time constraints [83]. Abnormal slowness is also manifest in the thought dynamics where MDD subjects tend to dwell longer on especially internally oriented thoughts whose abnormal slowness directly relates to the severity of the subjects’ rumination, e.g., brooding [82]. Moreover, slowness with decreased change can also be observed in emotions which show decreased changes over time in MDD [84].

Even action and perception are not exempted from the abnormal slowness in MDD. Action is signified by decreased change over time leading to psychomotor retardation [85–89]. Abnormal slowness is also manifest in visual perception where MDD subjects require a longer time to recognize the direction of dynamically moving visual gratings [90–93]. Finally, the perception of time is by itself featured by abnormal slowness and decreased speed in MDD subjects [90–93].

Together, these findings strongly support the idea that the dynamic reorganization on the neural level towards longer timescales and slower speed is accompanied by corresponding dynamic reorganization on the psychological level. ‘Neural slowness’ thus seems to translate into more or less corresponding ‘psychological slowness’. The exact mechanisms of such a relation of neural and psychological slowness remain yet unclear, though. One way to investigate that could be to establish a time series of both neural activity changes and psychological data on thoughts or emotions [84] which allows to measure their speed with the same measures as the autocorrelation window [84]. Again, as in the case of synchronization, one would expect analogous changes in the speed measures in both neural and psychological data.

### Co-occurrence of depressive symptoms—spatiotemporal “glue” and network approach

**Fig. 7:**
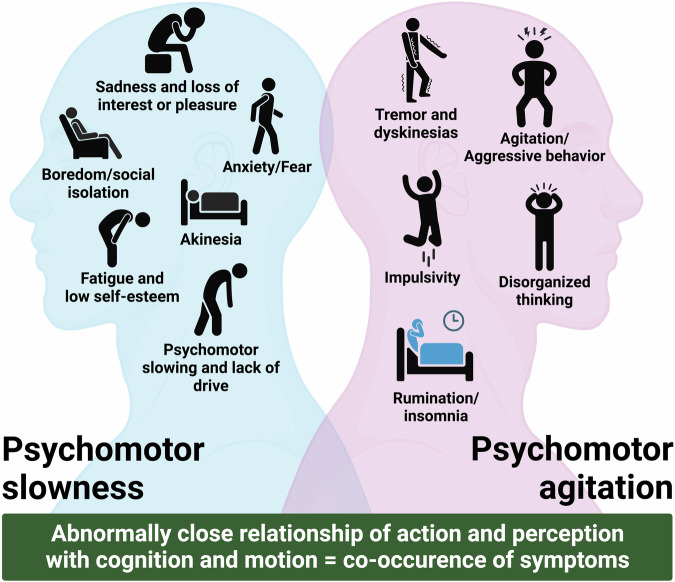
The co-occurrence of different psychopathological symptoms of psychomotor slowness and psychomotor agitation in major depressive disorder (MDD).

We saw that the global topographic and dynamic changes affect basically all psychological domains ranging from perception and action over emotion and cognition to even the sense of self and time. The assumption of multiple co-occurring psychopathological symptoms is in accordance with the current clinically used nosological systems like DSM-5 and ICD-11 which consider a certain number of psychopathological symptoms as diagnostically specific for a particular disorder category (Fig. 7). However, while a multitude of psychopathological symptoms is here indeed considered as necessary for the diagnosis of MDD, their systematic or intrinsic relationships are neglected. This systematic or intrinsic relationship manifests what we describe as the “co-occurrence of psychopathological symptoms” which we define by several features:

1. co-occurrence concerns the concurrent presence of multiple psychopathological symptoms usually stemming from different functional domains like affective, cognitive, motor, somatic-vegetative, sensory-perceptual, and interpersonal;
2. these psychopathological symptoms reflect abnormal balances and relationships between the different domains like increased cognition as in rumination typically co-occurring with decreased motor activity, e.g., psychomotor retardation;
3. these balances and relationships exhibit some systematic or intrinsic pattern as for instance increased cognition (rumination) co-occurring only with extremely negative mood, e.g., MDD, but not with an elevated positive mood, e.g., mania;
4. the different psychopathological symptoms including their systematic relationships can occur either simultaneously or sequentially during the course of an acute episode (or more chronic courses) as for instance psychomotor retardation in depression often precedes the acute mood symptoms in MDD;
5. the co-occurrence of different psychopathological symptoms may be deemed diagnostically specific for a particular syndrome (or dimension) (rather than a category) in a more or less cross- or trans-diagnostic (categorical) way as it is, for instance, the case in catatonia [94, 95], MDD [96] or SZ [97] (Fig. 1);

We now suggest that the systematic global topographic and dynamic changes on both neural and psychological levels provide the intrinsic or systematic relationships among the different psychopathological symptoms, e.g., their “glue”. Changes in the degree of unimodal transmodal synchronization and their disbalances in short long timescales relate to changes in the relationship of both perception and action with emotion and cognition. The respective associated psychopathological symptoms in these functional domains may thus stem from their underlying global topographic and dynamic disbalances on the neural level rather than from local deficits of their respective underlying regions, networks, or circuits including their timescales. The co-occurrence of the different psychopathological symptoms may then be characterized in a spatial or topographic way as manifest in their degree of simultaneity, e.g., synchronization, and their temporal or dynamic features, e.g., the sequence and fluctuations of their occurrence. The various psychopathological symptoms including their co-occurrence may thus be shaped in a primarily spatiotemporal way, that is, by the underlying global topographic dynamic changes in the brain. This amounts to what we recently introduced as “Spatiotemporal psychopathology” [95, 98–100].

Such spatiotemporal characterization of the psychopathological symptoms including their co-occurrence converges with the more psychological network approach (NA). Like the co-occurrence of psychopathological symptoms, the NA does not consider psychopathological symptoms as isolated atomistic entities but rather as inter-dependent as they are supposed to be constituted within a network; these networks and their causal interactions are assumed to be constitutive of these psychopathological symptoms [101, 102]. The different psychopathological symptoms can therefore be considered as ‘causal agents’ which, within the network, interact with each other in abnormal ways [103] – they are intrinsically related to each other amounting to what we describe as co-occurrence of psychopathological symptoms.

One key focus of NA is on MDD where various studies about its dynamics have been reported. Wichers et al. [104] investigated 6 MDD subjects with subjective reporting (‘experience sampling’) 3 times per day over 3-6 months. Measuring the ACW over the time series of these subjective data, they observed longer ACW with higher variance one month before the onset of an acute depressive episode; the prolongation of the ACW thus reflects an early “critical slowing down” (CSD). Analogous prolongation of the ACW in subjective data time series in MDD was also observed by others [84, 105, 106]. Finally, such increased slowing goes along with increased connectivity among different behavioral variables within the psychological network of MDD [104, 106]. Albeit tentatively, we suppose that such slowing down on the psychological and behavioral level relates to a more or less corresponding slowing down on the neural level, e.g., decreased speed in neural activity, for which there is indeed some empirical support as described above.

## Conclusion

MDD is characterized by a multitude of psychopathological symptoms, e.g., co-occurrence, whose underlying neural mechanisms remain yet unclear. We, reviewing recent findings, show that the brain’s global topography is shifted from unimodal sensory to transmodal associative regions in MDD. Next, we suggest two candidate mechanisms, the dynamic shift from shorter to longer timescales and changes in the excitation-inhibition balance, to mediate the abnormal unimodal transmodal topographic shift. Finally, we show how such topographic dynamic reorganization leads to the co-occurrence of the various MDD psychopathological symptoms featured by their intrinsic or systematic relationships. This requires that the current more local measures focusing on specific regions, networks, and frequencies be complemented by more global measures like the GS, global functional connectivity, and ACW (see Box 2 for details).

Together, we propose topographic and dynamic reorganization on both neural and psychological levels in MDD which is shared as the “common currency” of the brain and symptoms (Northoff et al. 2020a and b. This makes it possible for such “Topographic dynamic reorganization” to establish an integrated brain mind or better-integrated brain-symptom model of MDD symptoms. Taken in this way, it further develops and provides the basis for a genuinely topographic and dynamic characterization of psychopathological symptoms thus entailing what has been introduced as “Spatiotemporal psychopathology” (Northoff et al. 2023 [98, 99], Northoff and Hirjak [95], Northoff et al. 2016 [99]). This extends the recent ‘Resting state hypothesis of depression’ [45] and complements other models of depression (see Box 1).
