# Correction: Is depression a global brain disorder with topographic dynamic reorganization?

**DOI:** 10.1038/s41398-024-03022-7

**Published:** 2024-07-31

**Authors:** Georg Northoff, Dusan Hirjak

**Affiliations:** 1 Mind, Brain Imaging and Neuroethics Research Unit, The Royal’s Institute of Mental Health Research, University of Ottawa, Ottawa, ON Canada; 2 Department of Psychiatry and Psychotherapy, Central Institute of Mental Health, Medical Faculty Mannheim, University of Heidelberg, Mannheim, Germany; 3 German Centre for Mental Health (DZPG), Partner Site Mannheim, Mannheim, Germany

**Keywords:** Molecular neuroscience, Pathogenesis

Correction to: *Translational Psychiatry* 10.1038/s41398-024-02995-9, published online 5 July 2024

In the Acknowledgements section the following statement was missing from this article: ‘All figures (1–7) were created with BioRender.com.’

The original article has been corrected.

